# Supplementary material for: Asynchronous magnetic resonance elastography: Shear wave speed reconstruction using noise correlation of incoherent waves
Source: Magn Reson Med. 2022 Oct 27;89(3):990–1001. doi: 10.1002/mrm.29502 (PMC9792433; doi:10.1002/mrm.29502)
Supplement: Supplementary file 1 — DATA S1 MATLAB code used to generate the numerical simulations of Figure 1 [file MRM-89-990-s002.zip › k-Wave/helpfiles/reorderBinarySensorData.html]

reorderBinarySensorData :: Functions (k-Wave)


# reorderBinarySensorData

Reorder data from a binary sensor mask.

## Syntax

```
data = reorderBinarySensorData(data, reorder_index)
```

## Description

`reorderBinarySensorData` reorders sensor data returned by the k-Wave simulation functions (e.g., `kspaceFirstOrder2D` and `kspaceFirstOrder3D`) according to a given reordering index. This is useful when using a binary sensor mask in place of a Cartesian sensor mask with nearest neighbour interpolation (Cartesian sensor masks are not supported by the C++ codes).

Example syntax (assuming `sensor.record` is not defined)

```
% convert Cartesian sensor mask to binary sensor mask
[sensor.mask, ~, reorder_index] = cart2grid(kgrid, sensor.mask);

% run simulation
sensor_data = kspaceFirstOrder3DC(kgrid, medium, source, sensor)

% reorder output to order used in Cartesian sensor mask
sensor_data = reorderBinarySensorData(sensor_data, reorder_index);
```

If recording multiple outputs, then pass the individual outputs (e.g., `sensor_data.ux`) to `reorderBinarySensorData`.

## Inputs

|  |  |
| --- | --- |
| `data` | sensor data returned by the k-Wave simulation functions indexed as `(sensor_point_index, time_index)` |
| `reorder_index` | order of the Cartesian sensor points in the binary mask (this is returned by `cart2grid`) |

## Outputs

|  |  |
| --- | --- |
| `data` | reordered data according to the order of the sensor points defined in the original Cartesian sensor mask |

## See Also

`reorderSensorData`, `unmaskSensorData`
